# Direct measurement of the spin gaps in a gated GaAs two-dimensional electron gas

**DOI:** 10.1186/1556-276X-8-138

**Published:** 2013-03-25

**Authors:** Tsai-Yu Huang, Chi-Te Liang, Yang Fang Chen, Michelle Y Simmons, Gil-Ho Kim, David A Ritchie

**Affiliations:** 1 Department of Physics, National Taiwan University, Taipei 106, Taiwan; 2 School of Physics, University of New South Wales, Sydney, NSW, 2052, Australia; 3 School of Electronic and Electrical Engineering, Sungkyunkwan University, Suwon 440-746, South Korea; 4 Cavendish Laboratory, J. J. Thomson Avenue, Cambridge, CB3 0HE, UK

**Keywords:** Spin, g-factor, Disorder

## Abstract

We have performed magnetotransport measurements on gated GaAs two-dimensional electron gases in which electrons are confined in a layer of the nanoscale. From the slopes of a pair of spin-split Landau levels (LLs) in the energy-magnetic field plane, we can perform *direct* measurements of the spin gap for different LLs. The measured *g*-factor *g* is greatly enhanced over its bulk value in GaAs (0.44) due to electron–electron (e-e) interactions. Our results suggest that both the spin gap and *g* determined from conventional activation energy studies can be very different from those obtained by direct measurements.

## Background

With the growing interest in spin-based quantum computation and spintronic applications [1], there is an increasing need to understand and accurately determine critical parameters of the electron spin degree of freedom. It is well established that when measuring an electron spin in an external magnetic field *B*, it can either align parallel to or antiparallel to *B*. The energy difference between these two discrete states, also known as the spin gap or Zeeman splitting, is given by *gμ*_B_*B* where *g* is the Lande *g*-factor and *μ*_B_ is the Bohr magneton. It is worth mentioning that successful application of the wide range of possible spin-dependent phenomena requires effective techniques for the electrical injection of spin-polarized currents [2]. It has been demonstrated that a net spin current can be produced when

(1) $$g\mu_{B}B>\left(\mathrm{kT},\Gamma \right),$$

where *kT* and Γ are the thermal and level broadening, respectively [3].

For practical applications, it is highly desirable that the generation of the spin currents can be accomplished without requiring the use of extremely high *B*. Therefore, an accurate measurement of the spin gap and *g*-factor would allow one to ensure that only a moderate *B* is required so that Equation 1 holds. Moreover, the precise measurement of the *g*-factor [4] would shed light on the predicted divergence of spin susceptibility *χ* ∝ g m* and ferromagnetic ground state [5], where the system exhibits the unexpected metal-insulator transition [6]. Here m* represents the effective mass of electron (or hole). Given that the spin gap is the most important energy scale in any spin system and the *g*-factor is the central quantity characterizing the response of an electron or hole spin to an applied *B*, there have been many attempts to measure the spin gap in the literature. A standard method of obtaining the spin gap is to perform activation energy measurements at the minimum of the longitudinal resistivity $\rho_{\mathrm{xx}}\approx \exp\left(\frac{-\Delta_{s}}{2\mathrm{kT}}\right)$, where Δ_s_ is the spin gap [7]. However, such a measurement is rather restrictive as *ρ*_*xx*_ must be very low and has to vary over at least an order of magnitude as a function of *T*. Moreover, Δ_s_ has to be much greater than the thermal energy *kT* over the whole measurement range. Most importantly, activation energy measurements yield the ‘mobility gap’, the width of the localized states in the energy spectrum. This may be quite different from the real spin gap which corresponds to the energy difference between the two maxima densities of neighboring extended states [4,8].

In this paper, we report a method to directly measure the spin gaps in two-dimensional electron gases (2DEGs), in which the electrons are usually confined in layers of the nanoscale. We can change the applied gate voltage *V*_g_ to vary the electron density *n*_2D_ and hence the local Fermi energy *E* in our system. By studying the peak positions of *ρ*_*xx*_ at various *n*_2D_ and *B*, we can construct the Landau levels in the *E*-*B* diagram. As shown later, from the difference between the slopes of a pair of spin-split Landau levels in the *E*-*B* plane, we are able to measure the *g*-factors for different Landau level indices *n* in the zero disorder limit. We find that the measured *g*-factors (approximately 10) are greatly enhanced over their bulk value (0.44). Most importantly, our results provide direct experimental evidence that both the spin gap and *g*-factor determined from the direct measurements are very different from those obtained by the conventional activation energy studies. A possible reason is that our method is conducted in the zero disorder limit, whereas activation studies are performed under the influence of the disorder within the quantum Hall system.

In the integer quantum Hall effect (IQHE), when the spin of the 2DEG is taken into consideration, in the zero disorder limit each Landau level splits into two with the corresponding energy given by

(2) $$E=\left(n+\frac{1}{2}\right)\left(\hbar \omega_{C}\right)\pm \frac{1}{2}g\mu_{B}B$$

where *ω*_C_ is the cyclotron frequency, and *n* = 0, 1, 2, 3…, respectively. According to early experimental work [9], it was established that in 2D systems in a magnetic field the *g*-factor is greatly enhanced over its bulk value due to exchange interactions [10,11]. The precise measurement of the *g*-factor in 2D systems is a highly topical issue [4] since it has been predicted to be enhanced in strongly interacting 2D systems that exhibit the unexpected zero-field metal-insulator transition [6].

## Methods

### Experimental details

Magnetoresistance measurements were performed on three gated Hall bars (samples A, B and C) made from modulation-doped GaAs/Al_0.33_Ga_0.67_As heterostructures. For sample A, the structure consists of a semi-insulating (SI) GaAs (001) substrate, followed by an undoped 20-nm GaAs quantum well, an 80-nm undoped Al_0.33_Ga_0.67_As spacer, a 210-nm Si-doped Al_0.33_Ga_0.67_As, and finally a 10-nm GaAs cap layer. For sample B, the structure consists of an SI GaAs (001) substrate, followed by an undoped 20-nm GaAs quantum well, a 77-nm undoped Al_0.33_Ga_0.67_As spacer, a 210-nm Si-doped Al_0.33_Ga_0.67_As, and finally a 10-nm GaAs cap layer. Sample C is a modulation-doped GaAs/AlGaAs heterostructure in which self-assembled InAs quantum dots are inserted into the center of the GaAs well [12]. The following sequence was grown on an SI GaAs (001) substrate: 40-nm undoped Al_0.33_Ga_0.67_As layer, 20-nm GaAs quantum well inserted with 2.15 monolayer of InAs quantum dots in the center, a 40-nm undoped Al_0.33_Ga_0.67_As spacer, a 20-nm Si-doped Al_0.33_Ga_0.67_As, and finally a 10-nm GaAs cap layer. Because of the lack of inversion symmetry and the presence of interface electric fields, zero-field spin splitting may be present in GaAs/AlGaAs heterostructures. However, it is expected that the energy splitting will be too small (0.01 K) to be important in our devices [13]. For sample A, at *V*_g_ = 0 the carrier concentration of the 2DEG was 1.14 × 10^11^ cm^-2^ with a mobility of 1.5 × 10^6^ cm^2^/Vs in the dark. For sample B, at *V*_g_ = 0 the carrier concentration of the 2DEG was 9.1 × 10^10^ cm^-2^ with a mobility of 2.0 × 10^6^ cm^2^/Vs in the dark. The self-assembled InAs dots act as scattering centers in the GaAs 2DEG [12,14]; thus, the 2DEG has a mobility much lower than those for samples A and B. For sample C, at *V*_g_ = 0 the carrier concentration of the 2DEG was 1.48 × 10^11^ cm^-2^ with a mobility of 1.86 × 10^4^ cm^2^/Vs in the dark. Experiments were performed in a He3 cryostat and the four-terminal magnetoresistance was measured with standard phase-sensitive lock-in techniques.

## Results and discussion

**Figure 1:**
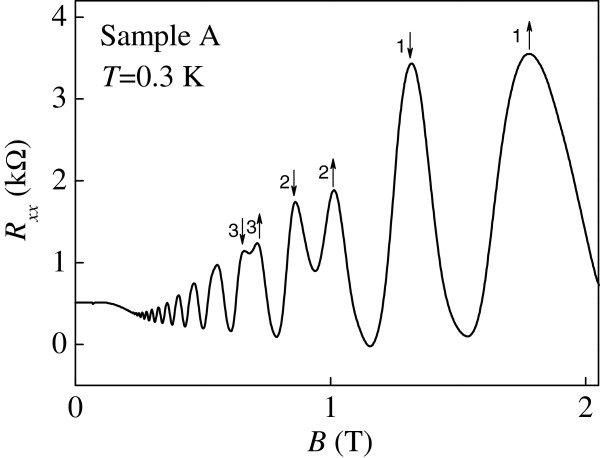
**Magnetoresistance measurements *R***_***xx ***_**( *B *) at *V***_**g **_**= -0.08 V for sample A at *T *= 0.3 K. **The maxima in *R*_*xx *_occur when the Fermi energy lies in the *n*th spin-split Landau levels as indicated by *n *= 3↓ and *n *= 3↑, *n *= 2↓ and *n* = 2↑, and *n *= 1↓ and *n *= 1↑, respectively.

**Figure 2:**
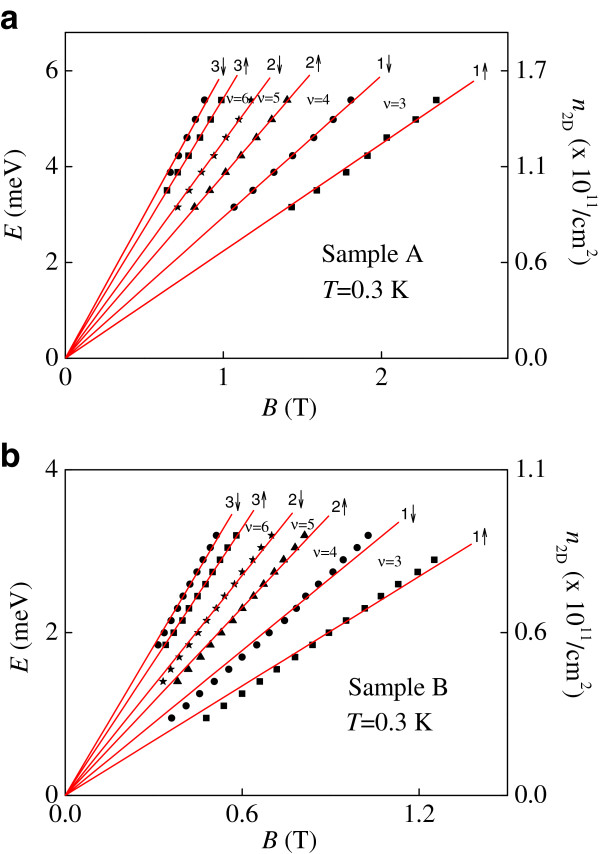
**The Local Fermi energy *E *and the corresponding 2D carrier density n**_**2D **_**for different Landau levels. **(**a**) Sample A and (**b**) sample B at *T *= 0.3 K. Circle, 3↓ and 1↓; square, 3↑ and 1↑; star, 2↓; triangle, 2↑.

**Figure 3:**
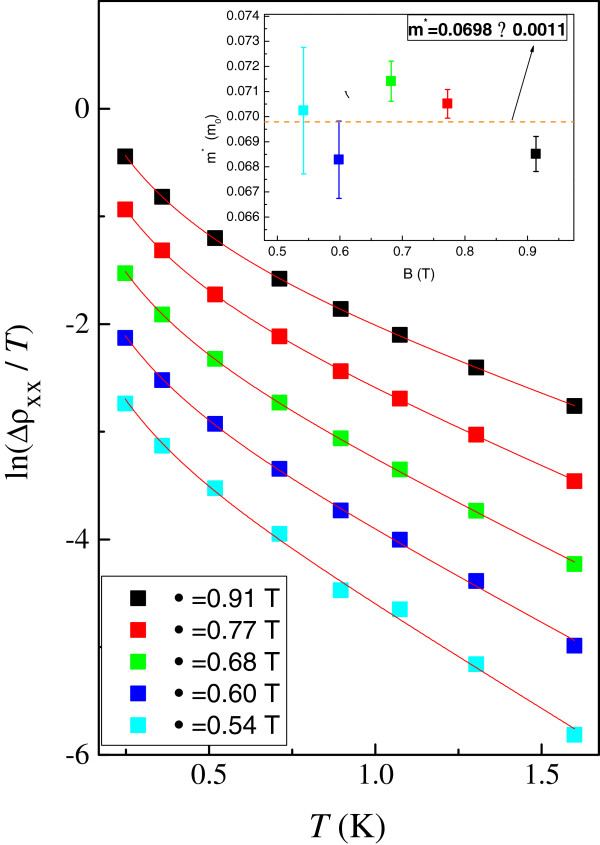
**Logarithm of the amplitudes of the oscillations. **The logarithm of the amplitudes of the oscillations divided by *T *ln(Δ*ρ*_xx _/ *T*) as a function of temperature at different magnetic field for sample C at *V*_g _= 0. The curves correspond to fits described by [18]. The inset shows the measured effective mass at different magnetic fields.

Figure 1 shows the four-terminal magnetoresistance measurements *R*_*xx*_ as a function of *B* at *V*_g_ = -0.08 V for sample A. When the Fermi level is centered at a Landau level, there exists a peak in *R*_*xx*_ as shown in Figure 1. By studying the evolution of the peaks in *R*_*xx*_ at different gate voltages (and hence *n*_2D_), we are able to locate the position of the Landau levels in the *n*_2D_-*B* plane. Figure 2a,b shows such results obtained from sample A and sample B, respectively. It is known that in the low disorder or high *B* limit, the filling factor of a resistivity (or conductivity) peak is given exactly by the average value of the filling factors of the two adjacent quantum Hall states [15]. This is equivalent to the situation when the Fermi energy coincides with a Landau level. It is worth pointing out that the peak position of magnetoresistance oscillations can be given by $n_{2D}=\frac{\mathrm{\nu eB}}{h}$, where *ν* is the Landau level filling factor. At first glance, the peak position does not depend on either the *g*-factor or the effective mass of the 2D system. However, as shown later, in our case the energy of the Landau levels can be considered directly proportional to the density via the free electron expression $E_{F}=\frac{\pi \hbar^{2}n_{2D}}{m^{*}}$[16], where m^*^ = 0.067 *m*_e_ in GaAs and *m*_e_ being the rest mass of a free electron. Then the effective mass should be considered when constructing the energy-magnetic field diagram. Here the oscillation of the Fermi energy is not considered. It may be possible that the effective mass of the 2DEGs will increase due to strong correlation effect [17]. In order to measure the effective mass of our 2DEG, we plot the logarithm of the resistivity oscillating amplitudes divided by temperature ln (Δ*ρ*_xx_ / *T*) as a function of temperature at different magnetic fields in Figure 3. Following the procedure described by the work of Braña and co-workers [18], as shown in the inset to Figure 3, the measured effective mass is very close to the expected value 0.067 *m*_e_. Therefore it is valid to use m^*^ = 0.067 *m*_e_ in our case. We can see that the Landau levels show a linear dependence in *B* as expected. At low *B* and hence low *n*_2D_, the slight deviation from the straight line fits can be ascribed to experimental uncertainties in measuring the positions of the spin-up and spin-down resistivity peaks.

In our system as the spin-split resistivity peaks are not observed at the *same* magnetic field, we need to describe the method of measuring the *g*-factors as follows. Equation 2 can be rewritten as

(3) $$E=\left[\left(n+\frac{1}{2}\right)\left(\frac{\hbar e}{m^{*}}\right)\pm \frac{1}{2}g^{*}\mu_{B}\right]B,$$

where we consider the effective Lande *g*-factor g^*^. We can see that Equation 3 corresponds to two straight line fits through the origin for a pair of spin-split Landau levels in the *E*-*B* plane as shown in Figure 2a,b. Such an approach was applied to a GaN-based 2DEG in our previous work [19]. We note that our method does depend on the exact functional form of the Landau band since the peak positions of the Landau level is only related to the carrier density in our system.

**Figure 4:**
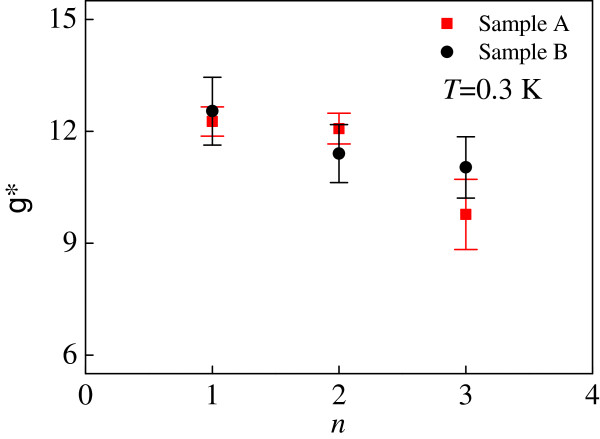
The measured g^* ^**as a function of Landau level index *n.*** The measured g^* ^as a function of Landau level index *n *for samples A and B at *T* = 0.3 K.

Let us now consider the region *ν* = 3 between the two linear fits corresponding to two spin-split Landau levels *n* = 1↓ and *n* = 1↑. According to Equation 3, the difference between the slopes of the spin-split Landau levels is given by g^*^Φ06Δ_*B*_*B*. Thus we are able to measure g^*^ for different Landau level indices (*n* = 1, 2, 3,…). In our system, the spin gap value is proportional to the magnetic field with good accuracy and corresponds to a constant g^*^ for a pair of given spin-split Landau levels. Figure 4 shows the measured g^*^ as a function of Landau level index *n* for samples A and B. In all cases, the measured g^*^ is greatly enhanced over its bulk value in GaAs (0.44). We ascribe this enhancement to exchange interactions. We suggest that the determined g^*^ is in the zero disorder limit since the positions of the spin-split Landau levels are located using Equation 2.

**Figure 5:**
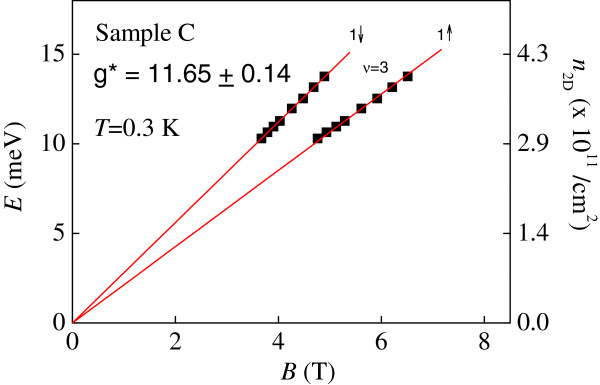
**Local Fermi energy *E *and the corresponding 2D carrier density n**_**2D**_**. **The local Fermi energy *E *and the corresponding 2D carrier density *n*_2D _for *n *= 1↓ and *n *= 1↑, Landau levels as a function of *B *for Sample C at *T *= 0.3 K.

It is worth mentioning that conventional activation energy studies are not applicable to our data obtained on sample A, sample B as well as the GaN-based 2DEG in our previous work [19]. The reason for this is that the values of the *R*_*xx*_ (and *σ*_*xx*_) minima are high; therefore, it is not appropriate to speak of electrons being thermally activated from the localized states to the extended states. In order to provide further understanding on the measurements of the spin gap, we have studied the slopes of the spin-split Landau levels in the *E*-*B* plane and have also performed conventional activation energy measurements on sample C over the *same* magnetic field range. Sample C is a more disordered device compared with samples A and B thus we can only perform measurements in the regime where the *ρ*_xx_ corresponding to a spin-split *ν* = 3 state is resolved. Figure 5 shows the evolution of the *n* = 1↓ and *n* = 1↑ resistivity peaks at different magnetic fields for sample C. From the difference between the two slopes of *n* = 1↓ and *n* = 1↑ spin-split Landau levels, the exchange-enhanced *g*-factor for the *n* = 1 Landau level is measured to be 11.65 ± 0.14, which is in close agreement with those obtained on a much higher mobility in samples A and B. We note that in a dilute GaAs 2DEG, the enhancement factor of *g* can decrease from about 6 to 3 as the density is reduced [20]. It may be possible that as our 2DEG density is considerably higher than those reported in the seminal work of Tutuc, Melinte, and Shayegan. Therefore we do not see such a trend in our system.

**Figure 6:**
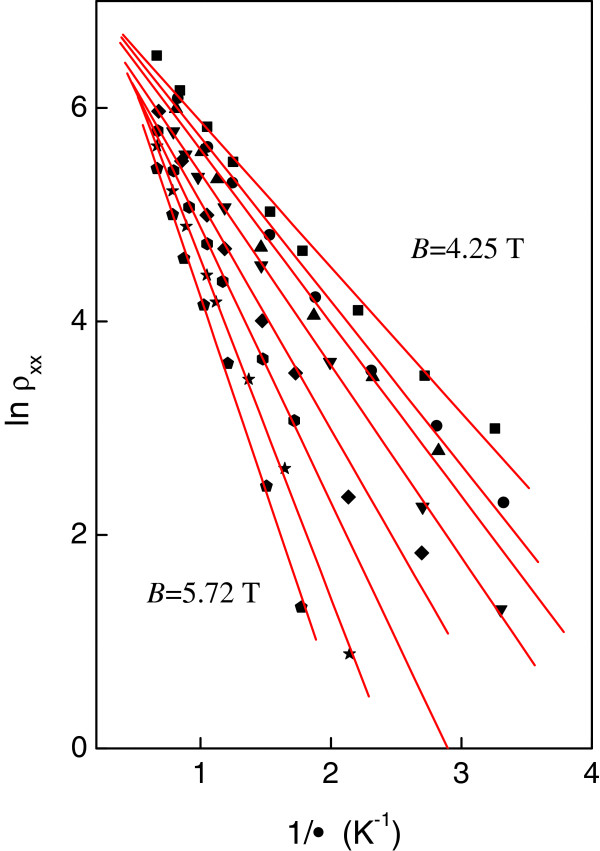
**Logarithm of *ρ***_***xx***_**( *B *)(*ν*** **= 3) versus the inverse of temperature 1/ *T *. **The logarithm of *ρ*_*xx*_(*B*)(*ν* = 3) versus the inverse of temperature 1/*T* at different gate voltages (and hence *B*) for sample C. From left to right: *B* = 5.72 (pentagon), 5.46 (star), 5.21 (hexagon), 4.97 (diamond), 4.70 (inverted triangle), 4.55 (triangle), 4.39 (heptagon) and 4.25 (square) *T*, respectively. The slopes of the straight line fits Δ_s _are shown in Figure 7.

**Figure 7:**
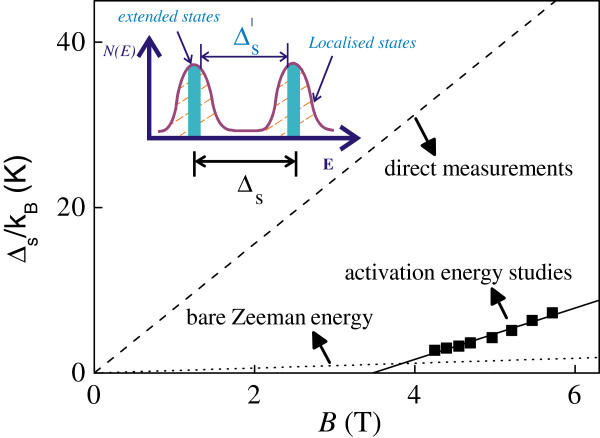
**The experimentally determined Δ**_**s**_**/*k***_**B **_**at various *B*. **The straight line fit is discussed in the text. The dotted line is the bare Zeeman energy assuming *g*_0 _= 0.44. The dashed line corresponds to the spin gap using the measured g^* ^= 11.65 by the direct measurements. The inset corresponds to a schematic diagram (density of states *N*(*E*) versus *E*) showing the spin gap *Δ*_s_ as a result of the activated behavior from the localized states (hatched areas) to the extended states (in blue). The spin gap in the zero disorder limit Δ_s_ is the energy difference between the neighboring peaks in *N*(*E*).

Let us now turn our attention to the activation energy measurements. Figure 6 shows ln (*ρ*_xx_) as a function of 1/*T* for eight different carrier densities while maintaining the filling factor at *ν* = 3 for sample C. The resistivity shows activated behavior $\rho_{\mathrm{xx}}\approx \text{exp}\left(\frac{-\Delta_{s}}{2\mathrm{kT}}\right)$. Figure 6 shows the activation energy Δ_s_ determined from a least-square fit to the experimental data shown in Figure 5. We can see that the spin gaps Δ_s_ drops approximately linearly to zero at a critical magnetic field *B*_c_ ~ 3.47 T. The spin gap is expected to have the form *Δ*_s_ = *g*_0_*μ*_B_*B* + *E*_ex_ = g^*^μ_B_*B*[12], where *E*_ex_ is the many-body exchange energy which lifts the *g*-factor from its bare value (0.44 in GaAs) to its enhanced value g^*^. Figure 7 shows that the measured Δ_s_ is greatly enhanced over the single particle Zeeman energy (shown in the dotted line), yielding g^*^ = 4.64 ± 0.30. Moreover, the exchange energy shows a roughly linear *B* dependence. The disorder broadening Γ_s_ can be estimated from the critical magnetic *B*_c_$\Gamma_{s}=\frac{\hbar }{\tau_{S}}=g^{*}\mu_{B}B_{C}$[12]. From this we obtain a quantum lifetime of Γ_s_ = 0.71 ps, in qualitative agreement with the value 0.40 ps obtained from the Dingle plot. For the low-field regime where Δ_s_ < Γ_s_, the many-body interactions are destroyed by the disorder, and there is no spin-splitting for the magnetic field less than *B*_c_. As shown in Figure 7, the ‘spin gap’ measured by the conventional activation energy studies is very different from that measured by the direct measurements (shown in the dashed line). This is consistent with the fact that activation energy studies yield a mobility gap which is smaller than the real spin gap in the spectrum. Moreover, the measured by studying the slopes of the *n* = 1 spin-split Landau levels is approximately 2.4 times larger than that determined from the activation energy studies. Our data shows that both the spin gaps and g^*^ measured by the activation energy studies are very different from those determined from direct measurements. A possible reason for this is that there exists disorder within 2D system which is indispensable to the observation of the IQHE. The direct measurements are performed in the zero disorder limit. On the other hand, in the activation energy studies, the disorder within the quantum Hall system must be considered. As shown in the inset of Figure 7, the spin gap in the zero disorder limit is the energy difference between neighboring peaks in the density of states *N*(*E*) which is larger than the energy spacing between the edges of the localized states given the finite extended states. We suggest that further theoretical studies are required in order to obtain a full understanding of our results on the spin gaps and g^*^.

## Conclusions

In conclusion, we have performed direct measurements of the spin gaps in gated GaAs 2DEGs by studying the slopes of spin-split Landau levels in the energy-magnetic field plane. The measured *g*-factor is greatly enhanced over its bulk value (0.44). Since disorder exists in any experimentally realized system, conventional activation energy studies always measure the mobility gap due to disorder which is different from the real spin gap as shown in our results. As the spin gap is one of the most important energy scales and governs the electron spin degree of freedom, our experimental results provide useful information in the field of spintronics, spin-related phenomena, and quantum computation applications.

## Competing interests

The authors declare that they have no competing interests.

## Authors’ contributions

TYH and CTL performed the measurements. CTL, YFC, and GHK coordinated the projects. MYS and DAR grew the samples. TYH, YFC, and CTL drafted the paper. All authors read and approved the final manuscript.
